# A framework for designing medical devices resilient to low-resource settings

**DOI:** 10.1186/s12992-021-00718-z

**Published:** 2021-06-22

**Authors:** Davide Piaggio, Rossana Castaldo, Marco Cinelli, Sara Cinelli, Alessia Maccaro, Leandro Pecchia

**Affiliations:** 1 School of Engineering, University of Warwick, Coventry, CV4 7AL UK; 2 Institute of Computing Science, Poznań University of Technology, Piotrowo 2, 60-965 Poznań, Poland; 3 Department of Information Engineering, University of Padova, 35131 Padova, Italy

**Keywords:** Medical device design, Low-resource settings, Delphi survey, Resilient medical devices, Contextual design, MCDA

## Abstract

**Background:**

To date (April 2021), medical device (MD) design approaches have failed to consider the contexts where MDs can be operationalised. Although most of the global population lives and is treated in Low- and Middle-Income Countries (LMCIs), over 80% of the MD market share is in high-resource settings, which set de facto standards that cannot be taken for granted in lower resource settings. Using a MD designed for high-resource settings in LMICs may hinder its safe and efficient operationalisation. In the literature, many criteria for frameworks to support resilient MD design were presented. However, since the available criteria (as of 2021) are far from being consensual and comprehensive, the aim of this study is to raise awareness about such challenges and to scope experts’ consensus regarding the essentiality of MD design criteria.

**Results:**

This paper presents a novel application of Delphi study and Multiple Criteria Decision Analysis (MCDA) to develop a framework comprising 26 essential criteria, which were evaluated and chosen by international experts coming from different parts of the world. This framework was validated by analysing some MDs presented in the WHO Compendium of innovative health technologies for low-resource settings.

**Conclusions:**

This novel holistic framework takes into account some domains that are usually underestimated by MDs designers. For this reason, it can be used by experts designing MDs resilient to low-resource settings and it can also assist policymakers and non-governmental organisations in shaping the future of global healthcare.

**Supplementary Information:**

The online version contains supplementary material available at 10.1186/s12992-021-00718-z.

## Background

According to the World Bank and the World Health Organisation (WHO), at least half of the world population had no access to essential health services in 2017 [1]. In fact, Arasaratnam et al. [2] affirmed that only 13% of the global population accounts for 76% of the global MD use, suggesting inequitable healthcare access in favour of higher resource settings. The latter also preside over the MD market, 80% of which is ruled by the USA, Europe and Japan [3]. These countries set and represent a de facto standard and can rely on medical settings that conform to international standards of quality, which should not be taken for granted in low-resource settings. This can be one of the reasons why in lower-income settings up to 70–90% of donated MDs [2, 4, 5] are non-operational or broken. Indeed, this often relates to the majority of available devices, for example 80% of the MDs of Sub-Saharan Africa are donated [6].

Low-resource settings, in particular, are characterised by generally poor environmental and operational conditions. In fact, healthcare is typically delivered in locations, which are not fit for purpose (e.g., dust, vermin, humidity, high temperatures, etc.) [6, 7]. The literature highlights the main challenges for healthcare provision in low-resource settings to be: lack of funding, insufficient essential MDs [7–10], lack of specialised doctors [7–11], lack of biomedical engineers and technicians (who can maintain or repair MDs), and deficiency of spare parts/consumables [7–10]. Further, the situation and needs can change significantly between city centres and the periphery. In 2007, Malkin [12] identified that capital cost, lack of spare parts and lack of consumables were the three main barriers to the introduction of healthcare technology in hospitals in low-resource settings. All these factors negatively influence the efficacy, safety and durability of MDs.

In the 2010s, some studies started relating this situation to a design problem. For instance, Aranda et al. [13], similarly to Kortum et al. [14], stated that healthcare facilities are in poor conditions in terms of basic infrastructure due to designers’ failure. Aranda et al. [11] also asserted that these issues could be efficiently tackled with a well-planned, user-driven and contextualised design. Similarly, to manufacture safe, efficient and effective MDs, it is essential to define both the user requirements and the context/environment of use, which in some cases is not considered as part of the design process [15].

In the literature, there have been attempts to identify the crucial criteria for the design of MDs resilient to low-resource settings [2, 5, 13, 16]. Arasaratnam et al. [2] gave an overview of the past trend, better known as “glocalisation”. This term, not to be confused with the frequently misused term “globalisation”, arises from the combination of the latter with the term “localisation”. It is used to describe a phenomenon of contextualisation of some products or services, universally distributed but adjusted/modified to accommodate the need of the user or consumer of local markets. Glocalisation was a typical trend of the industry, which removed some features from high-tech products that were designed for more developed countries to make them accessible to low-resource settings. Nowadays, it is widely recognised that this trend is not sufficient to adjust medical technologies to low-resource settings. Thus, the new trend is so-called “frugal innovation” [17]. The study by Aranda et al. [13], relying on experts’ views, identified some essential criteria in the durability, robustness, cost of ownership over time and simplicity of use. The same criteria were pointed out by Nimunkar et al. [16] along with accuracy, reliability, size, weight, materials, power requirements, ease of manufacture, language barriers, availability of facilities and population dynamics. The latter also agreed with the widely shared belief that special considerations should be made when designing MDs for developing countries. Similar results were obtained by Gauthier et al. [5] through a literature review and an analysis of state-of-the-art MDs designed for low-resource settings. Beyond the already mentioned criteria, Gauthier et al. [5] also suggested further criteria such as appropriateness, functionality, spare parts, personnel, management, and public policy. Moreover, the authors also asserted that the primary focus should be on affordability, durability, and materials [5].

Interestingly, in the ‘90s Adler et al. [18] presented and described the application of some military standards to the design of MDs for military use. This did not seem to receive as much attention as the current (2021) trend followed by the Information Technology (IT) industry, which is adopting the Ingress Protection (IP) Code (International Electrotechnical Commission (IEC) standard 60,529) and military standards (specifically, MIL-STD-810H) to design and manufacture “ruggedised” devices, which are sturdier and more resilient to harsh working conditions. Nonetheless, there is no overall agreement on a framework including a set of criteria that supports the design of MDs for low-resource settings, outlining the key considerations and desirable properties.

The aim of this study is to contribute to bridging this research gap by systematically developing a general framework that could guide the design of MDs for low-resource settings. In particular, this study aims to identify, classify and weigh the essential criteria to be considered while designing MDs resilient to low-resource settings. The framework was initiated starting from the MDs presented in the WHO compendium of innovative health technologies for low-resource settings [19].

Our goal was pursued inductively, i.e., by inferring the general set of essential criteria from observations/data. A triangulation of qualitative and quantitative methods was used. In fact, as Pope et al. affirmed [20], the former can complement the latter when used as an essential preliminary step to quantitative research and can also be supplementary information to further validate the quantitative results. Such methods, which will be thoroughly described in the paper, include: focus groups, field studies, content analysis for drafting and validating a set of essential criteria, pilot tests, semi-structured interviews, Delphi survey, and multi-criteria decision analysis (MCDA) for developing and validating a questionnaire, and analysing the results. As a result, the final version of the questionnaire (see Additional File 1) was issued to a pool of experts to complete. As a result, a set of essential criteria was obtained and used to develop a framework, i.e., a multi-criteria decision system for designing MDs resilient to low-resource settings.

This work could lay the basis for the creation of novel and contextualised international standards, which could empower LMICs, enabling them to enforce import restrictions according to the World Trade Organization Agreement on Trade-Related Aspects of Intellectual Property Rights (WTO-TRIPS), and creating “an indigenous medical equipment manufacturing capability” [12]. Thus, this would force manufacturers to design and manufacture products in line with the new consensus standard to give them the opportunity to market their products in LMICs.

## Methods

**Fig. 1:**
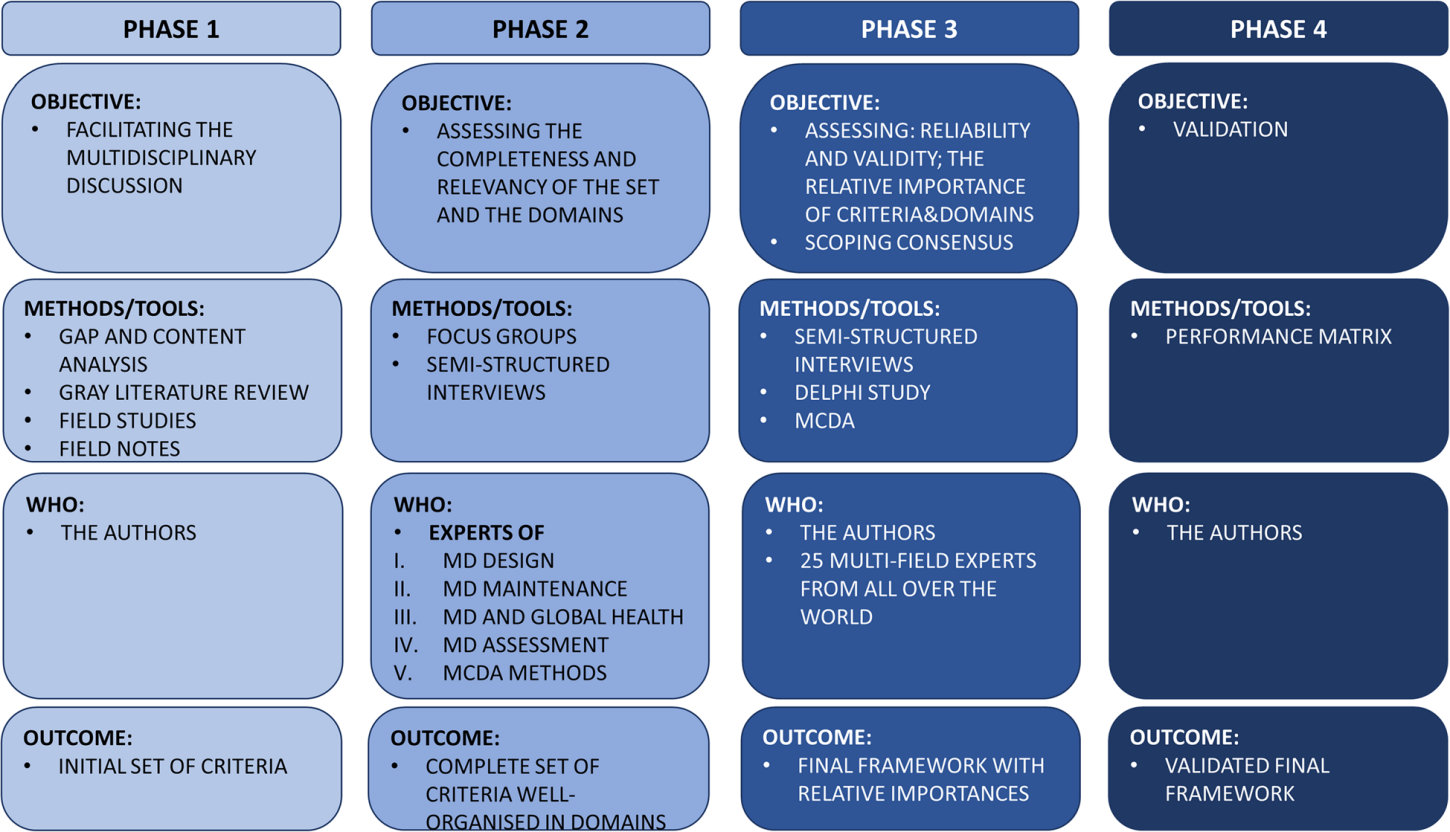
A summary of the phases, their objectives, methods, people involved and outcomes

The inductive method, which implies the generation of theories starting from numerous observations [21], was used to develop the set of essential criteria. In particular, a triangulation of qualitative and quantitative methods was used to develop a framework of criteria for the design of MDs resilient to low-resource settings and for its validation through the convergence of information [20, 22]. Figure 1 summarises the project phases along with their main objectives, methods, people involved and outcomes. In particular, purposive sampling [20, 23] was used for the methods that required sampling. The latter is a non-random technique that consists of the researcher deliberately choosing participants, based on the information that they can provide relying on their knowledge or experience [24].

### Criteria identification

**Fig. 2:**
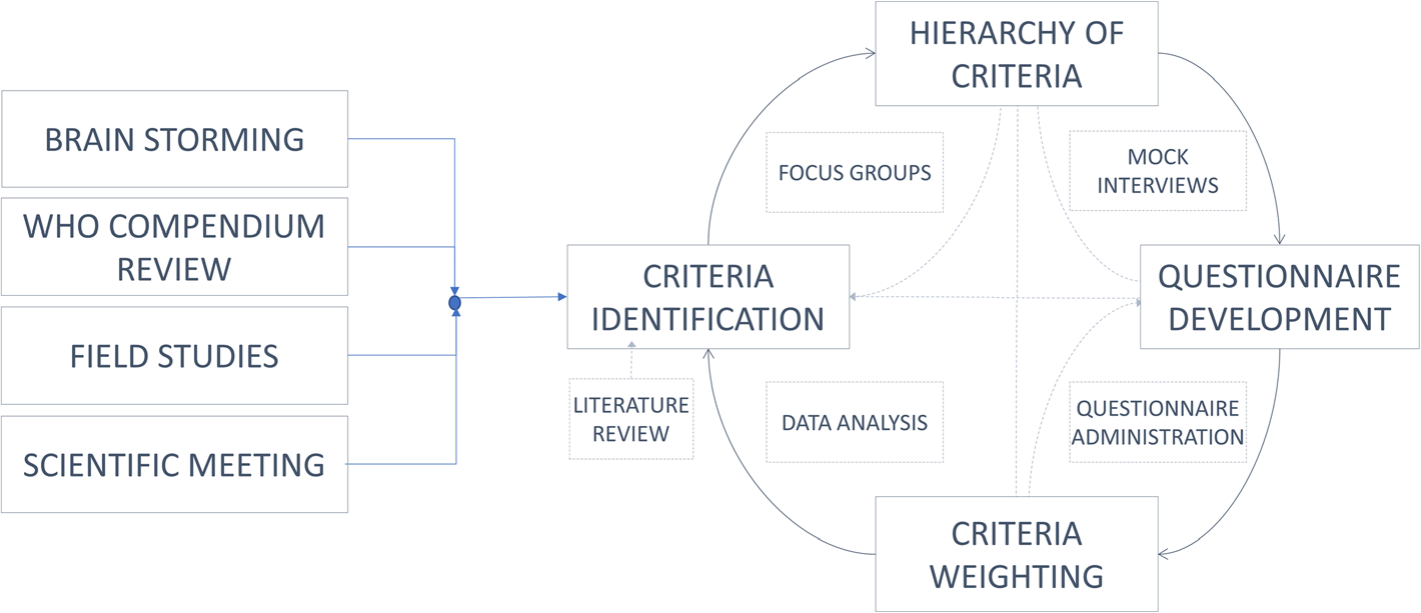
Block diagram of the study process and methods used

The identification, classification and weighting of the fundamental criteria for the design of MDs for low-income settings were achieved with a series of nested closed loops involving relevant scholars and experts (see Fig. 2) from five continents. All the steps presented in Figs. 1 and 2 are interdependent.

After analysing the criteria for the design of medical locations and MDs, essential criteria for the resilient design for low-resource settings were identified by reviewing systematically the existing scientific literature. These initial criteria and the rationale of this study were presented at the AfricaHealth Conference (Johannesburg, June 2016) and discussed in a focus group with biomedical and clinical engineers from 15 Sub-Saharan Africa countries.

In November 2016, the essential criteria were reviewed during a 3-day meeting organised in Warwick in collaboration with the IFMBE HTAD division (http://htad.ifmbe.org/) working group for the preparation of guidelines of the Health Technology Assessment of MDs [25]. During the meeting, 14 experts from 8 nations and 1 WHO representative participated in a focus group to analyse the interdependencies among working environments and MDs safety and effectiveness [15]. During this focus group, the essential criteria were enriched with experts’ feedback and a grey literature review on low-resource settings, design of MDs and medical locations. Particular attention was given to the WHO compendium of innovative health technologies for low-resource settings [19]. Two authors (DP, SC) analysed the compendium and reported the MDs that are supposedly resilient to harsh environments. The recurrent design criteria were noted, narrowed down and finally enriched with the experts’ views.

### Hierarchy of criteria

The identified criteria were clustered in meaningful domains defining a hierarchy framework. The hierarchy was piloted in Africa with several field studies, each followed by an additional focus group. In May 2017 and January 2018, two field analyses were carried out in Benin performing visual inspections and testing of MDs and medical locations [6] to evaluate the relevance and the suitability of the essential criteria set. The former field study was followed by a focus group with experts from Sub-Saharan Africa during the AfricaHealth2017 (Johannesburg, South Africa). The latter field study was followed by a focus group held in Addis Ababa, Ethiopia, during the kick-off meeting of the IFMBE Working Group on BME in Africa, in February 2018 [26]. Additionally, in 2019 two extra field studies were carried out in Benin and Uganda [7].

### Survey

Once the set of essential criteria was consolidated, one investigator (DP) created the first draft of the questionnaire to confirm the set of criteria, explore consensus among scholars and domain experts, and weight criteria with relative importance.

The survey was pilot-tested in two stages. Firstly, it was administered to the participants of the first focus group in Warwick for possible disambiguation and correction of content and language. Secondly, it was tested with in-person semi-structured mock interviews at the International Union for Physical and Engineering Sciences in Medicine (IUPESM) World Congress (Prague, June 2018), recruiting scholars not involved in the previous steps. In particular, during the IUPESM Congress, one of the authors (DP) conducted semi-structured interviews and collected the answers of biomedical and clinical engineering experts, whose feedback helped us refine the questionnaire and enrich it with one additional criterion. Eventually, another focus group was held to review the criteria and to create the final version of the questionnaire. The final questionnaire was sent with a bilingual email invitation, linking to two online questionnaires, one in English and one in French, to improve the reachability of African experts from francophone countries. One reminder was sent after 1 month to the panellists who did not reply.

Due to our extensive network of institutions, responders were identified amongst the ones collaborating with the IFMBE Health Technology Assessment, Clinical Engineering divisions and speakers selected by the WHO for the sessions on MD design and management in LMICs of the Fourth WHO Global Forum on Medical Devices. Finally, authors of relevant scientific papers were invited, which were independent of the above institutions. Responders were recommended based on their experience with health technologies, including design, development, testing, implementation and maintenance of MDs. The study received full ethical approval by the University of Warwick Ethical BSREC Committee (REGO-2018-2283).

### Delphi survey design

Named after the famous oracle at Delphi, the Delphi study is one of the several consensus methods available [27]. It is an iterative multistage process that aims to elicit and reinforce experts’ opinions via group consensus with a series of structured questionnaires, which the experts fill in. This can be conducted in two main ways. The questionnaire can be anonymous, i.e. the respondents’ identity is private and they cannot see another respondent’s answers; or it can be blinded, i.e. the respondents’ identity is only known by the investigators and they are still “blind” to others’ answers. This allows every participant to freely express their own opinions, minimises the “bandwagon effect” (i.e. the phenomenon in which people tend to do something just because other people do it, regardless of their own beliefs or opinions [28]) and prevents the authority or reputation of some participant from dominating others [29]. In our study, we conducted a blind online Delphi survey, which allowed us to reach expert panellists all around the world. Our study aimed to obtain consensus merging the panellists’ different opinions through group dynamics, rather than to achieve statistical power. Therefore, a priori power analysis to calculate the minimum sample number of panellists was not evaluated for this study. However, we referred to the existing literature that suggests that a panel should include 10 to 18 experts [27].

### Delphi process

The questionnaire was composed of 42 questions, organised in 9 sections: 1 regarding the responder professional experience, 7 weighting the importance of design criteria clustered in different domains and 1 to weigh the relative importance of such domains. Each section closed with an open question to gather suggestions about new criteria, if any. All the acronyms of the questionnaire were duly explained. Definitions were given for some of the criteria. In fact, given the respondents are experienced in the domain of MD design with a special focus on low-resource settings, we assumed that well-established concepts in this domain needed no further explanation (i.e. maintenance frequency, maintenance cost, the need for consumables or spare parts, etc.). Conversely, for the criteria that were not deemed self-explanatory, examples/definitions (please see Additional File 1) were added in brackets in the questions of the questionnaire as in the following examples:

- For “reliance on medical location air”, examples were given such as “filtering, temperature, humidity, etc.”
- For “end users’ background”, examples were given such as “doctors, nurses, biomedical engineering technicians”
- For “ease of use”, examples were given such as “the medical device requires a high specialised personnel, any operator can use it, or it is so intuitive that anyone can use it” etc.)

The questions regarding the design criteria were asked with a repetitive formula, namely “*What is the importance of considering* “CRITERION 1” *during the design of MD which will be used in low-resource settings?*” Such questions had six possible answers, converted to an integer number based on a 5 steps Likert-type scale, i.e., “Very low” (1), “Low” (2), “Middle” (3), “High” (4), “Very high” (5), “Do not know” (““). A new criterion would be included in a subsequent Delphi round if proposed by at least 30% of the respondents [25]. Regarding the iteration stopping criteria, we decided not to iterate the questionnaire if all of the criteria and domain reached consensus defined as follows. For all the criteria, the first and third quartiles and their relative distances ‘Δ’, i.e., the interquartile ranges, were calculated in order to rank the criteria consensus with a “Sufficient” range (2 ≥ Δ ≥ 1), “Fair” range (1 ≥ Δ > 0) or a “Full consensus” range (Δ = 0).

### Data analysis

#### Reliability

When performing a survey, it is crucial to assess the reliability of the responses. Low reliability negatively affects the validity of the results [30]. Reliability measures how the criteria are part of the same domain and are, thus, relevantly measuring the same overarching concept. This is fundamental since experienced experts may also provide inconsistent answers due to tiredness or distraction. Following the procedure previously employed in other studies [31, 32], reliability was estimated through ordinal alpha and the use of polychoric matrices. The latter are optimum tools for analysing the reliability in case of ordinal data (such as Likert-scale based questionnaires) because Cronbach alpha is based on the assumption of continuous data [33] and could lead to underestimation [34]. Ordinal alpha scale, in particular, ranges from 0 to 1, with values over 0.7 being “Acceptable” [31].

Polychoric correlation matrices and the consequent ordinal alphas were calculated in R (version 3.5.1) with the functions “polychoric()” and “alpha()” for each domain.

As suggested in [35], once the ordinal alphas were calculated, internal consistency was rated according to the values reported in Table 1. Then, the alpha drop was also calculated for the domains that showed low internal consistency. The latter is a measure of how the alpha would change if a criterion of the domain was dropped. This helps identify the problematic elements of the questionnaire, if any.

**Table 1:** The values of alpha and their interpretation

Alpha	Internal Consistency
α ≥ 0.9	Excellent
0.8 ≤ α < 0.9	Good
0.7 ≤ α < 0.8	Acceptable
0.6 ≤ α < 0.7	Questionable
0.5 ≤ α < 0.6	Poor
α ≤ 0.5	Unacceptable

#### Validity

Validity is an essential component of survey evaluation; in fact, it tests whether the scale is measuring what it is intended to measure [31]. In this case, validity was tested during the pilot test, in which interviewees were asked for additional comments and also during the Delphi survey. Each domain contained a final question asking to suggest criteria, their ranking, and possible comments. New criteria would be added if suggested by at least 30% of the panellists.

#### Ranking

In order to find the final importance of each criterion derived from the answers to the questionnaire, the relative index was calculated. The Relative Index (RI) is defined by the following eq. [31]:

$$ RI=\sum \limits_{i=1}^5\frac{w_i{f}_i}{N} $$

Where *w*_*i*_ is the weighting factor calculated by dividing the rating score by the highest score (i.e., 5), *f*_*i*_ is the frequency of the responses and *N* is the total number of responses. Once the relative indices were calculated, the criteria were sorted in decreasing order and divided into subclasses according to their importance, namely Fundamental, Important and Relevant.

#### Correlations among criteria

Correlations among criteria in the same domain were assessed with Goodman and Kruskal’s gamma, which was calculated with the following eq. [31]:

$$ \gamma =\frac{SOP- IOP}{SOP+ IOP} $$

Where *SOP* stands for “Same Order Pair” and *IOP* for “Inverse Order Pair”. Gamma (*γ*) ranges from − 1 to 1 and in this case values greater than 0.5 or lower than − 0.5 were considered to highlight a strong correlation, whose significance depends on the *p*-value. In this case, we selected a p-value of 0.05.

### Validation and use of the framework

Once identified, the relevant criteria were combined into a framework that could be used to help inform the design of MDs resilient to low-resource settings.

Subsequently, a protocol was developed to test this framework of criteria with a subset of MDs and to show its possible use. All the criteria were reported and divided into two sets: discriminatory and non-discriminatory criteria. Three criteria were added a posteriori of our field studies, under the “Reliance on external factors” domain.

A total of 8 MDs was randomly extracted from the WHO compendia of innovative health technologies for low-resource settings, 4 from the 2014 version [19] and 4 from the 2017 version [36].

After analysing the selected devices, information regarding each of the criteria was used to score them with the help of a performance matrix (similarly to the concept of Pugh matrix [37]). The criteria from the cost and lifetime domains, i.e., non-discriminatory criteria, were excluded from this analysis because they cannot be scored qualitatively. A qualitative three point-based measurement scale was used. Adopting the well-known traffic light colour code, red was used for the worst cases, yellow for the intermediate performance and green for the best case. Consequently, two trends were evaluated:

1. Which criteria feature mainly green cells (i.e., a good assessment) irrespective of the device? In this case, only the criteria reaching a good assessment in at least 5 out of the 8 MDs were selected.
2. Which of the selected MDs resulted efficient (i.e. criteria prevalently coded as green), which averagely efficient (i.e. criteria prevalently coded as yellow) and which not really efficient (i.e. criteria prevalently coded as red)?

## Results

### Focus group and pilot

The final version of the hierarchy of essential criteria for the design of MDs resilient to low-resource setting working conditions resulted from the second focus groups and brainstorming session. Specifically, the criteria were grouped into seven main domains as follows:

1. User type
2. Health technology management (HTM)
3. Design
4. Reliance on external factors
5. Material
6. Cost
7. Lifetime

Moreover, the piloting phase led to the addition of one criterion, namely “the capability of the user to understand the technical and clinical impact of the technology”.

### Delphi survey

#### Characteristics of Panellists

We invited 56 professionals, with a background spanning from biomedical engineering to clinical engineering and public health, to participate in the final survey, and an overall 25/56 (44.6%) answered the questionnaire. The 25 panellists represented 19 countries, 7/25 (28%) of them represented Europe, 12/25 (48%) Africa, 2/25 (8%) North America, 2/25 (8%) East Asia, 1/25 (4%) Central America, and 1/25 (4%) South America. As far as Africa is concerned, the involved countries were the Democratic Republic of Congo, Ethiopia, Tanzania, Mozambique, Benin, South Africa, Ghana, Kenya and Burundi. The fields of expertise of the respondents are broad and summarised Table 2. Since some respondents had more than one area of expertise, the different areas of expertise were counted as separate entries. The average years of expertise of the responders were 18.6 years and the standard deviation 12.2 years.

**Table 2:** A summary of the areas of expertise of the panellists. The numbers are out of 29 respondents as more than one respondent stated more than one area of expertise. The different areas of expertise were counted as separate entries

Area of Expertise	Number (%)
Biomedical engineering	9/29 (31.0%)
Clinical engineering	6/29 (20.7%)
Medical devices & Instrumentation design	7/29 (24.1%)
Life cycle management of MDs	4/29 (13.8%)
Health technology assessment	2/29 (6.9%)
Other	1/29 (3.5%)

#### Consensus on criteria

The median, Interquartile Range (IQR) (i.e. the range containing 50% of the data, obtained by subtracting the first quartile from the third quartile) and its interpretation, and the number of panellists for each recommendation are shown in Additional File 2. All the criteria reached the consensus threshold.

### Reliability analysis

Six out of seven domains showed a high internal consistency (see Table 3). This means that all the identified domains included a coherent and relevant set of criteria. Only the User Type domain showed a low ordinal alpha (0.58). In this case, we also performed further analysis to study how the ordinal alpha would change if one criterion would be omitted. As a result, the ordinal alpha reached an acceptable value of 0.76 by not considering the easiness of use.

**Table 3:** Summary of the reliability analysis

Statistics	User Type	HTM	Design	Reliance on ext. factors	Material	Cost	Lifetime
**Number of replies**	24	25	25	20	25	25	25
**Reliability of scale**	0.58	0.92	0.7	0.9	0.96	0.9	0.84
**Interpretation**	Poor	Excellent	Acceptable	Excellent	Excellent	Excellent	Good
**# of criteria**	4	6	3	5	2	3	2
**Changes applied after pilot survey**	1 criterion added	–	–	–	–	–	–

### Validity

The pilot study helped refine how questions were presented and one criterion was added during the piloting. Furthermore, although the respondents to the final survey suggested some additional criteria through the open questions, none of these was supported and shared by more than 3/18 respondents (16.7, < 30% cut-off).

### Ranking and correlations

**Fig. 3:**
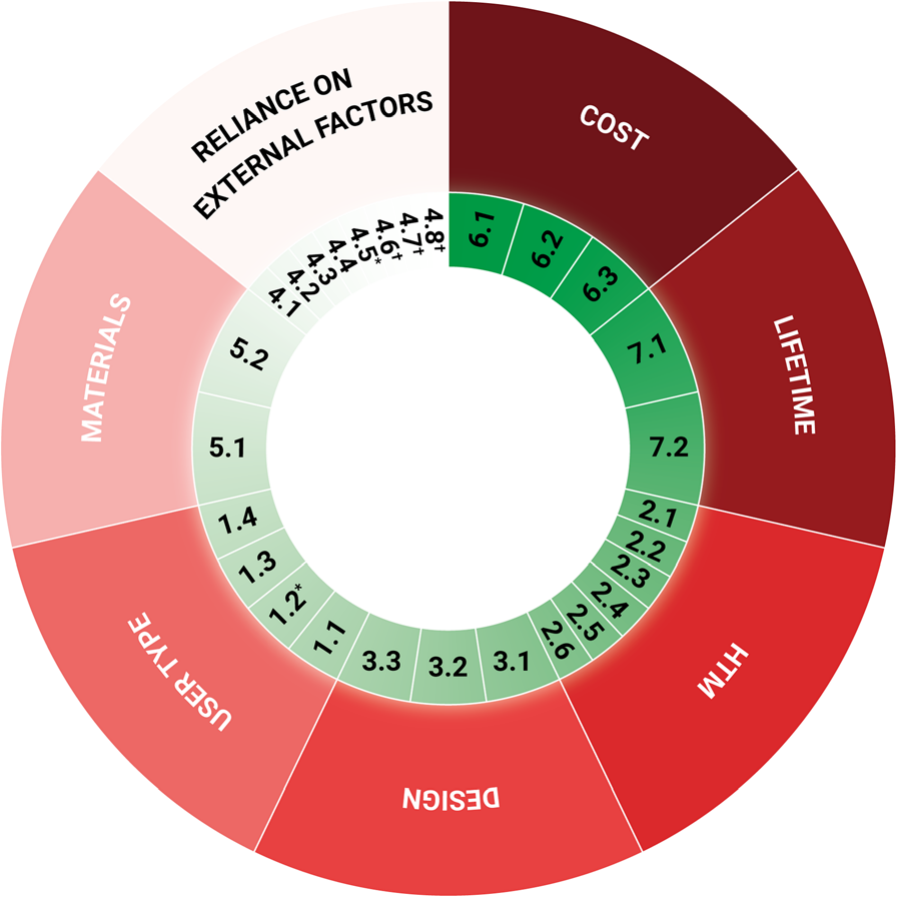
The final framework containing the domains and the criteria ranked according to the level of importance. The domains, although approximately sharing the same importance, are presented in descending order of importance starting from cost, clockwise. Each criterion is presented in the same descending order within each domain, too. *Criterion 1.2 is the criterion that was excluded after reliability/internal consistency analysis. *Criterion 4.5 is the criterion that was excluded because deemed of Medium importance. †Criteria 4.6, 4.7 and 4.8 are the criteria added a posteriori of our field studies and they are not currently ranked. The legend for the criteria in this figure can be found in Table 4

All the calculated relative indexes were over 0.5, implying that all the criteria raised a great interest in the panellists. Also, all of the indexes were very close to each other, highlighting the great importance of each domain, which in decreasing order were 0.888 for cost, 0.848 for lifetime, 0.84 for HTM, 0.832 for design, 0.824 user type, 0.824 for materials and 0.792 for reliance on external factors (see Fig. 3).

**Fig. 4:**
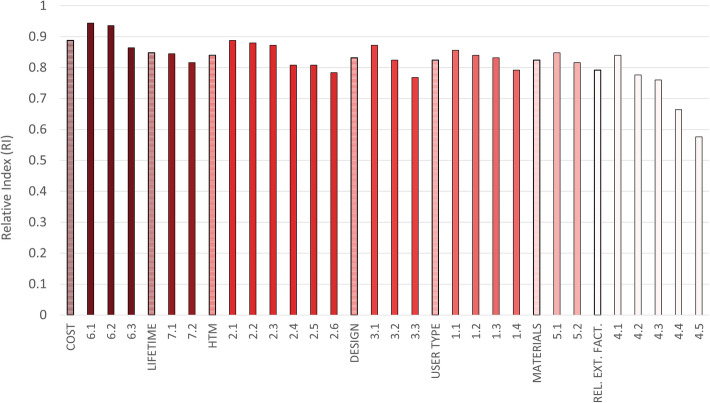
The relative indexes are reported for each criterion, grouped by domain. The first column of each group is the relative index of the domain itself. The full name of the criteria could not be reported for brevity. The legend is presented in Table 4

In particular, the next sections show the results grouped by domain (see Table 4 and Fig. 4).

**Table 4:** The correlations of the criteria grouped by domain. Correlation was reported only if “strong” (i.e., gamma greater than 0.5) and if the respective *p*-value was less than 0.05. The number specified in the correlation column refers to that of the correlated criteria. The “+” indicates a positive correlation, the “-“a negative correlation. †Criteria 4.6, 4.7 and 4.8 are the criteria added a posteriori of our field studies and they are not currently ranked. For this reason, also correlations are not calculated nor reported (NA)

Criteria	Correlation
6. Cost	
6.1 Maintenance costs	6.2,6.3(+)
6.2 Running costs	6.1(+)
6.3 Initial cost	6.1(+)
7. Lifetime	
7.1 Lifetime of MD parts/components	7.2 (+)
7.2 MD lifetime	7.1(+)
2. HTM	
2.1 Need for consumables	2.2,2.3,2.4,2.5(+)
2.2 Need for spare parts	2.1,2.3,2.4,2.5(+)
2.3 Installation requirements	2.1,2.2,2.4,2.5(+)
2.4 Maintenance complexity	2.1,2.2,2.3,2.5,2.6(+)
2.5 Maintenance frequency	2.1,2.2,2.3,2.4(+)
2.6 Compatible consumables/spare parts	2.4(+)
3. Design	
3.1 Portability, compactness, robustness	3.2(+)
3.2 Limiting the number of components/spare parts	3.1(+)
3.3 Reusability	–
1. User type	
1.1 End users’ background	1.4(+)
1.2 Easiness of use	–
1.3 Training needs	1.4(+)
1.4 User’s understanding of the technical and clinical impact	1.1(+)
5. Material	
5.1 Durability of the material	5.2(+)
5.2 Robustness of the material	5.1 (+)
4. Reliance on external factors	
4.1 Reliance on power sources	4.2,4.3,4.4(+)
4.2 Reliance on water distribution	4.1,4.3,4.4(+)
4.3 Reliance on medical location air	4.1,4.2,4.4,4.5(+)
4.4 Need for sample preparation	4.1,4.2,4.3,4.5(+)
4.5 Understanding/stating the dependence of the MD from the medical location characteristics	4.3,4.4(+)
4.6 Resilience to dusty environments^†^	NA
4.7 Resilience to high-temperature environments^†^	
4.8 Resilience to high-humidity environments^†^

#### Cost

Maintenance cost (6.1), running costs (6.2) and initial cost (6.3) are “Fundamental”, although the initial cost is seen as slightly less important with respect to the first two.

#### Lifetime

Both the lifetime of the MD (7.2) and its parts/components (7.1) are “Fundamental” and correlated. The lifetime of a MD depends on the functionality of its parts, in fact, more components are likely to cause more breakdowns.

#### HTM

The need for consumables (2.1) is number one in this domain and is positively correlated with the need for spare parts (2.2), the installation requirements (2.3), the maintenance complexity (2.4) and frequency (2.5). This stresses recurring problems reported in the literature, in which the lack of spare parts, consumables, and functional maintenance are very common [8–10].

#### Design

Portability, compactness, robustness (3.1) and limiting the number of components/spare parts (3.2) are ranked as “Fundamental” and are correlated. Portability and compactness are greatly influenced by the number of components or spare parts. Limiting the latter would improve the durability of a functional device because fewer components are likely to reduce breakdowns.

#### User type

The end-users’ background (1.1) ranks at the top of this domain and is positively correlated with the users’ understanding of the technical and clinical impacts (1.4). The latter is dependent on the former: a solid background is key to understanding the technical and clinical impacts. As regards the easiness of use (1.2), negatively correlated with the end-user background (1.1) (although not significantly), it was discarded because of how it negatively affected the overall reliability of the user type domain. In this case, this variable could be re-assessed by the panellists to see if the results may change.

#### Materials

Both the durability (5.1) and the robustness (5.2) of the material are “Fundamental” and are correlated.

#### Reliance on external factors

Reliance on power sources (4.1) comes first in this domain, followed by the reliance on water distribution (4.2), the reliance on location air (4.3) and the need for sample preparation (4.4), with which it is correlated. All these are essential to the efficient and effective use of certain MDs. Regarding the understanding and stating the dependence of the MD from the medical location characteristics (4.5) indicated a low RI value of 0.576. Therefore, the Delphi study presented a consensus of the criterion being “Medium” importance and the authors decided to discard it. Three additional criteria were added a posteriori of our field studies, i.e., resilience to dusty environments (4.6), resilience to high-temperature environments (4.7), and resilience to high-humidity environments (4.8).

### Validation and use of the framework

The protocol (see Additional File 3) to test the framework includes an information table with the qualitative scoring of the MDs. The criteria selected only as informative and not discriminatory of a technology belong to the cost and lifetime domain. This further division was introduced because the comparison for such domains could only be made if there were references to use. For example, if someone were trying to compare two MDs with similar functions, they would need a MD of reference in order to consider the cost and life domains discriminatory. In this scenario, it would then be possible to compare the costs and the lifetime of one of the MDs compared to the one of reference and consequently assign those values such as “low”, “medium”, or “high”.

The 3 criteria added a posteriori are “Resilience to dusty environments”, “Resilience to high-temperature environments” and “Resilience to high-humidity environments”.

According to the traffic light rating system adopted, poor performances were marked as red, medium performance as yellow, and good performance as green.

For example, as regards the “End users’ background”, red would denote the fact that the technology could only be used by medical doctors, yellow by any health care professionals and green by lay users. Or as regards “Reliance on power sources”, red would denote the fact that the technology relies only on the power supply, yellow on the power supply and batteries, and green on the latter and solar panels. Applying this process to one of the selected MDs, i.e. the pulse oximeter, to make further examples, we can notice how it scores yellow for “End users’ background”, as lay users are not enlisted among possible end-users. Such a device also scores green for “Training needs”, as no further training is required, and scores red (also the missing data are classed as red) for resilience to dusty, high temperature and humidity environments due to the manufacturer not providing any information about these problematic environments.

The selected MDs were namely: low-cost computed tomography scanner, pulse oximeter, anaesthesia delivery for low-resource settings, mobile-enabled non-invasive measure-through motion and low perfusion pulse oximeter, electrocardiogram handheld digital, phototherapy for jaundice, external fixation for bone fracture and white blood cell counting system.

Only 9 out of the 21 discriminatory criteria performed generally well (predominantly green cells) and they belonged to different domains: installation requirements, maintenance frequency and complexity, portability, compactness and robustness, limiting the number of components/spare parts, reusability, reliance on water distribution, reliance on medical location air and need for sample preparation.

The 3 MDs that resulted efficient are the two pulse oximeters and the external fixation for bone fracture. The 4 MDs, whose efficiency resulted medium, are the electrocardiogram, the phototherapy device and the white blood cell counting system. Finally, the computed tomography scanner and the anaesthesia machine resulted not very efficient.

## Discussion

This study aimed to respond to the urgent need for a comprehensive framework for the design of MDs resilient to low-resource settings and to propose a possible solution. To date (April 2021), MD design frameworks have been proven ineffective when using the MDs outside of “their bubble”, i.e. settings that have more resources and some guaranteed standards that are not readily available in low-resource settings [38]. The results of this study show the timeliness and importance of such a topic. In fact, the international experts’ views were elicited through MCDA, which was proven to aid decision making processes, knowledge production and consumption [39], being able to capture all the various dimensions of the evaluation problem [40], enhancing the quality of decisions, allowing them to be more rational and efficient and transparent [39, 41, 42], also in the relevant emerging technology and biomedical fields [43].

From the results, it emerged that the experts in different fields shared their opinions and agreed on most of the criteria. A particular criterion had a slightly lower response rate 20/25, with 5 panellists who replied, “Do not know”. The authors think that this criterion, i.e. Understanding/stating the dependence of the MD from the medical location characteristics (i.e. Group 0,1,2), was underestimated probably because of a general non-expertise in such a specific topic.

Correlations are essential to understand how criteria are interrelated. In fact, they give an understanding of the trends that characterise the set of criteria. Positive correlation between criteria A and B means that the higher the importance of criterion A is also followed by higher importance for criterion B. The opposite is true in the case of negative correlation. This type of information is useful to inform the design of the MDs, since the criteria with positive correlations need to be jointly taken into account to guarantee the successful design and development of a MD. For example, the domain of cost, running costs are positively correlated with maintenance costs, but not to the initial costs. This confirms that the respondents consider these two kinds of costs interrelated. Hence, they need to be jointly considered when designing a MD.

From the results, it is clear that even if all the domains share a similar relative index of importance, the resulting priorities are a comprehensive representation of the current state of the art (as of 2021) regarding the design of MDs for low-resource settings. In particular, the domain of cost comes first, and the domain of the reliance on external factors comes last. However, within the domain of cost, the operating and maintenance costs resulted to be slightly prioritised in respect to the initial cost. Although capital costs can be high for some equipment, the operating or maintenance costs are often underestimated. Malkin [12] had concluded that for a MD to be effective and economical at point of purchase is not sufficient for a technology to help low-resource settings, highlighting common misconceptions, such as the belief that the capital cost of a technology is always the primary barrier.

Indeed, the cost is crucial, but the underestimation of the reliance on external factors or of the user type (i.e. the last but two) is dangerous. These factors are the ones that may severely influence the functional operation of MDs, as it has also been reported in the literature, and that leads to the so-called “contextualised design”.

For this reason, as mentioned before, after their latest field studies in Benin and Uganda, the authors of this paper decided to include three new criteria that underline critical challenges for low-resource settings, i.e. the presence of dusty environments, high temperatures and humidity. Once again, this highlights further the importance of these factors that are often overlooked.

In the authors’ opinion, the results from this study are a relevant representation of the current (2021) approach of MD designers. Clearly, there is a need to change the way of approaching MD design if we want to support global health without inequalities across the countries.

This study is the first of its kind to use a triangulation of qualitative and quantitative methods to obtain a comprehensive and effective framework for the design of MDs resilient to low-resource settings. This framework is further proof of the need for a change and adaptation both of the existing international standards and the MD design criteria. The authors believe that the approach presented by Adler [18] and currently utilised by the IT industry, i.e. the use of the IP code standard and military standards (MIL-STD-810H) is the future of MD design. The harsh conditions typical of low-resource settings, in fact, could be simulated through apposite tests for humidity, temperature, pressure and others adapted from the military world.

Furthermore, any researcher or designer involved in MD design could potentially use this framework either to check if an existing design is compliant or to start a new innovative and/or conceptual design.

The validation of this framework proved that the protocol to follow for assessing a MD is quite simple and does not require external expert inputs. Any person with a biomedical engineering background should be able to complete the framework and straightforwardly assess the technology. The validation also confirmed that the domain of reliance on external factors is often underestimated and resulted in obtaining majority of yellow and red scores.

### Limitations of the study

As already mentioned, some of the criteria were classified as informative, because, in order to use such domains for comparisons, there is a need for extra references, which can be investigated in case someone wanted to compare different options of the same kind of technology.

In addition, some of the scales are only available as qualitative, as semi-quantitative scales would need to be investigated further with ad-hoc studies, and there are several “-“, meaning that no information is available to score some MDs. The latter are data gaps that should be filled to get a more realistic understanding of the performances. At the time of writing, missing data/gaps were assigned the worst-case colour (i.e., red), using a precautionary approach.

Finally, the validation carried out is a valid internal validation of the framework. However, an additional external validation/testing could preferably be performed as well.

## Conclusions

The aim of this study was to highlight current (2021) challenges and gaps in the design process of MDs, to highlight the urgency in addressing such gaps and to create a framework for contextual and user-driven design of MDs. Such a framework will facilitate design and production of MDs which will perform correctly and efficiently in any country of the world, irrespective of the available income and/or resources. Although frameworks exist, such as the frugal framework of Aranda et al. [13], the focus here is the aspects of MDs on which the designer has a potential influence.

The advantages of the framework presented in this paper are the fact that it emphasises the urgency of taking into consideration particular realities and the technical tool it offers. Such a tool, in fact, can be one of the first steps towards this inclusive approach. Nonetheless, it is pivotal to underline that the authors’ invitation to take contextual realities into account should not be misinterpreted as “relativism”, because trying to defend and safeguard differences will lead to failing to see any common prospect.

Rather, this framework fosters glocalisation, as per Bauman’s definition [44]: i.e. the preservation of individual identities within a complex system. In fact, far from dividing the world into different parts, each characterised by the richness of its peculiarities (none being superior to the others), we could reaffirm Augé’s theory of the “Non-place”, i.e. an effectively inclusive space where each context finds its citizenship [45].

To conclude, this framework could encourage and inform the creation of novel contextualised international standards, which would empower LMICs by enforcing import restrictions according to the WTO-TRIPS, by fostering the local production of medical equipment and by obliging MD manufacturers to comply with this new consensus standard if they wanted to market their products in LMICs.

Starting from this paper, further international political actions should follow to bring forward design frameworks based on the best available evidence and real users’ needs elicited by experts, in order to drive the real change of the existing norms, regulations and standards.

## Supplementary Information

**Additional file 1.** is a copy of the final version of the questionnaire.

**Additional file 2.** is a Word file (.docx) and is a table containing the design criteria with median, interquartile range, its interpretation and the number of panellists.

**Additional file 3.** is an Excel file (.xlsx) and is the tool that was used for validating the framework and that can be used for assessing other technologies.

## Data Availability

The datasets used and/or analysed during this study are available from the corresponding author on reasonable request.
