# Supplementary material for: A framework for designing medical devices resilient to low-resource settings
Source: Global Health. 2021 Jun 22;17:64. doi: 10.1186/s12992-021-00718-z (PMC8220789; doi:10.1186/s12992-021-00718-z)
Supplement: Supplementary file 1 — Additional file 1. is a copy of the final version of the questionnaire. [file 12992_2021_718_MOESM1_ESM.pdf]

Medical device design criteria for low-resource settings

This short (10 min) survey aims to identify essential criteria that, in your opinion, should be considered while designing Medical Devices (MDs) that will be used in lower income settings. This survey aims to explore which elements should be considered during the design of MDs to make them more resilient to operational conditions that in lower-income settings are more challenging than in higher-income ones (e.g., lack of specialised staff, health technology management issues, poor supply chain etc.).

Your name has been suggested by your colleagues and IFMBE AC members, which consider you as one of the leading experts for medical device assessment, maintenance or regulation.

The answers will be anonymized and used by IFMBE (http://ifmbe.org) studies and in research studies within the Applied Biomedical Signal Processing and Intelligent eHealth Lab (https://warwick.ac.uk/fac/sci/eng/research/grouplist/biomedicaleng/abspie) at the University of Warwick, led by Dr Leandro Pecchia.

Your answers will not be disclosed and your participation to this study will remain confidential. Your email is collected only to share with you the final results of this survey. Since this is an electronic survey, taking part in the survey and transmitting your answers online, we assume that you give your consent and agree that we can use the information you provide us with for the purposes of our study.

This study was approved by the Biomedical & Scientific Research Ethics Committee (BSREC) at the University of Warwick (REGO-2018-2283).

*Required

1. **Email address** *

2. **In which nation do you work?** *

3. **What's your field of expertise?** *

4. **How many years of experience do you have in the field?** *

5. **USER TYPE: What is the importance of considering the END USERS' BACKGROUND (e.g., doctors, nurses, biomedical engineering technicians etc.) during the design of MD which will be used in low resource settings?** *

   Mark only one oval per row.

	Very low	Low	Medium	High	Very high	Do not know
Select an option						

6. **USER TYPE: What is the importance of considering the EASINESS OF USE (e.g., the MD requires a high specialized personnel, any operators can use it, or it is so intuitive that anyone can use it etc.) during the design of MD which will be used in low resource settings? ***

Mark only one oval per row.

	Very low	Low	Medium	High	Very high	Do not know
Select an option	◯	◯	◯	◯	◯	◯

7. **USER TYPE: What is the importance of considering the TRAINING NEEDS (e.g., the training required to become skilled with this technology) during the design of MD which will be used in low resource settings? ***

Mark only one oval per row.

	Very low	Low	Medium	High	Very high	Do not know
Select an option	◯	◯	◯	◯	◯	◯

8. **USER TYPE: What is the importance of considering the USER'S UNDERSTANDING OF THE TECHNICAL AND CLINICAL IMPACT of the technology during the design of MD which will be used in low resource settings? ***

Mark only one oval per row.

	Very low	Low	Medium	High	Very high	Do not know
Select an option	◯	◯	◯	◯	◯	◯

9. **Do you suggest any other essential criterion/a for the USER TYPE domain? If yes, please include each below and rate its importance between brackets [e.g., "criteria 1 (Very low)" or "criteria 1 (High), criteria 2 (Medium), … "] ***

____
____
____
____
____

10. **HTM (Health Technology Management): What is the importance of considering the INSTALLATION REQUIREMENTS during the design of MD which will be used in low resource settings? ***

Mark only one oval per row.

	Very low	Low	Medium	High	Very high	Do not know
Select an option	◯	◯	◯	◯	◯	◯

11. **HTM: What is the importance of considering the MAINTENANCE FREQUENCY during the design of MD which will be used in low resource settings? ***

Mark only one oval per row.

	Very low	Low	Medium	High	Very high	Do not know
Select an option	◯	◯	◯	◯	◯	◯

12. **HTM: What is the importance of considering the MAINTENANCE COMPLEXITY during the design of MD which will be used in low resource settings? ***

Mark only one oval per row.

	Very low	Low	Medium	High	Very high	Do not know
Select an option	◯	◯	◯	◯	◯	◯

13. **HTM: What is the importance of considering the need for CONSUMABLES during the design of MD which will be used in low resource settings?** *

Mark only one oval per row.

	Very low	Low	Medium	High	Very high	Do not know
Select an option	◯	◯	◯	◯	◯	◯

14. **HTM: What is the importance of considering the need for SPARE PARTS during the design of MD which will be used in low resource settings?** *

Mark only one oval per row.

	Very low	Low	Medium	High	Very high	Do not know
Select an option	◯	◯	◯	◯	◯	◯

15. **HTM: How important is the fact that consumables/spare parts are not necessarily from the same manufacturer (e.g., COMPATIBLE CONSUMABLES/SPARE PARTS CAN BE USED)?** *

Mark only one oval per row.

	Very low	Low	Medium	High	Very high	Do not know
Select an option	◯	◯	◯	◯	◯	◯

16. **Do you suggest any other essential criterion/a for the HTM domain? If yes, please include each below and rate its importance between brackets [e.g., "criteria 1 (Very low)" or "criteria 1 (High), criteria 2 (Medium), … "]** *

____

____

____

____

____

17. **DESIGN: What is the importance of considering PORTABILITY, COMPACTNESS, ROBUSTNESS during the design of MD which will be used in low resource settings?** *

Mark only one oval per row.

	Very low	Low	Medium	High	Very high	Do not know
Select an option	◯	◯	◯	◯	◯	◯

18. **DESIGN: What is the importance of LIMITING THE NUMBER OF COMPONENTS/SPARE PARTS during the design of MD which will be used in low resource settings?** *

Mark only one oval per row.

	Very low	Low	Medium	High	Very high	Do not know
Select an option	◯	◯	◯	◯	◯	◯

19. **DESIGN: What is the importance of considering the REUSABILITY during the design of MD which will be used in low resource settings?** *

Mark only one oval per row.

	Very low	Low	Medium	High	Very high	Do not know
Select an option	◯	◯	◯	◯	◯	◯

20. **Do you suggest any other essential criterion/a for the DESIGN domain? If yes, please include each below and rate its importance between brackets [e.g., "criteria 1 (Very low)" or "criteria 1 (High), criteria 2 (Medium), … "]** *

21. **RELIANCE ON EXTERNAL FACTORS: What is the importance of considering the RELIANCE ON POWER SOURCES during the design of MD which will be used in low resource settings?** *

Mark only one oval per row.

	Very low	Low	Medium	High	Very high	Do not know
Select an option	⬭	⬭	⬭	⬭	⬭	⬭

22. **RELIANCE ON EXTERNAL FACTORS: What is the importance of considering the RELIANCE ON WATER DISTRIBUTION during the design of MD which will be used in low resource settings?** *

Mark only one oval per row.

	Very low	Low	Medium	High	Very high	Do not know
Select an option	⬭	⬭	⬭	⬭	⬭	⬭

23. **RELIANCE ON EXTERNAL FACTORS: What is the importance of considering the RELIANCE ON MEDICAL LOCATION AIR (e.g. filtering, temperature, humidity, number of air-changes per hour) during the design of MD which will be used in low resource settings?** *

Mark only one oval per row.

	Very low	Low	Medium	High	Very high	Do not know
Select an option	⬭	⬭	⬭	⬭	⬭	⬭

24. **RELIANCE ON EXTERNAL FACTORS: What is the importance of UNDERSTANDING/STATING THE DEPENDENCE OF THE MD FROM THE MEDICAL LOCATION CHARACTERISTICS (e.g. limited use in group 0, 2 or 3) during the design of MD which will be used in low resource settings?** *

Mark only one oval per row.

	Very low	Low	Medium	High	Very high	Do not know
Select an option	⬭	⬭	⬭	⬭	⬭	⬭

25. **RELIANCE ON EXTERNAL FACTORS: What is the importance of considering the NEED FOR SAMPLE PREPARATION (when applicable) during the design of MD which will be used in low resource settings?** *

Mark only one oval per row.

	Very low	Low	Medium	High	Very high	Do not know
Select an option	⬭	⬭	⬭	⬭	⬭	⬭

26. **Do you suggest any other essential criterion/a for the RELIANCE ON EXTERNAL FACTORS domain? If yes, please include each below and rate its importance between brackets [e.g., "criteria 1 (Very low)" or "criteria 1 (High), criteria 2 (Medium), … "]** *

27. **MATERIAL: What is the importance of considering the ROBUSTNESS OF THE MATERIAL used during the design of MD which will be used in low resource settings?** *
Mark only one oval per row.

	Very low	Low	Medium	High	Very high	Do not know
Select an option	◯	◯	◯	◯	◯	◯

28. **MATERIAL: What is the importance of considering the DURABILITY OF THE CHOSEN MATERIAL during the design of MD which will be used in low resource settings?** *
Mark only one oval per row.

	Very low	Low	Medium	High	Very high	Do not know
Select an option	◯	◯	◯	◯	◯	◯

29. **Do you suggest any other essential criterion/a for the MATERIAL domain? If yes, please include each below and rate its importance between brackets [e.g., "criteria 1 (Very low)" or "criteria 1 (High), criteria 2 (Medium), … "]** *

30. **COST: What is the importance of considering the INITIAL COST during the design of MD which will be used in low resource settings?** *
Mark only one oval per row.

	Very low	Low	Medium	High	Very high	Do not know
Select an option	◯	◯	◯	◯	◯	◯

31. **COST: What is the importance of considering the MAINTENANCE COST during the design of MD which will be used in low resource settings?** *
Mark only one oval per row.

	Very low	Low	Medium	High	Very high	Do not know
Select an option	◯	◯	◯	◯	◯	◯

32. **COST: What is the importance of considering the RUNNING COSTS during the design of MD which will be used in low resource settings?** *
Mark only one oval per row.

	Very low	Low	Medium	High	Very high	Do not know
Select an option	◯	◯	◯	◯	◯	◯

33. **Do you suggest any other essential criterion/a for the COST domain? If yes, please include each below and rate its importance between brackets [e.g., "criteria 1 (Very low)" or "criteria 1 (High), criteria 2 (Medium), … "]** *

34. **LIFETIME: What is the importance of considering the MD LIFETIME during the design of MD which will be used in low resource settings?** *

Mark only one oval per row.

	Very low	Low	Medium	High	Very high	Do not know
Select an option						

35. **LIFETIME: What is the importance of considering the LIFETIME OF MD PARTS/COMPONENTS during the design of MD which will be used in low resource settings?** *

Mark only one oval per row.

	Very low	Low	Medium	High	Very high	Do not know
Select an option						

36. **Do you suggest any other essential criterion/a for the LIFETIME domain? If yes, please include each below and rate its importance between brackets [e.g., "criteria 1 (Very low)" or "criteria 1 (High), criteria 2 (Medium), … "]** *

37. **Please rate the importance of each domain for low resource settings** *

Mark only one oval per row.

	Very low	Low	Medium	High	Very high	Do not know
USER TYPE						
HTM						
DESIGN						
RELIANCE ON EXTERNAL FACTORS						
MATERIALS USED TO BUILD THE MD						
COST						
LIFETIME						

Send me a copy of my responses.
