# Supplementary material for: A framework for designing medical devices resilient to low-resource settings
Source: Global Health. 2021 Jun 22;17:64. doi: 10.1186/s12992-021-00718-z (PMC8220789; doi:10.1186/s12992-021-00718-z)
Supplement: Supplementary file 2 — Additional file 2. is a Word file (.docx) and is a table containing the design criteria with median, interquartile range, its interpretation and the number of panellists. [file 12992_2021_718_MOESM2_ESM.docx]

Additional file 1. Design criteria with median, interquartile range, its interpretation and the number of panellists.

| Criteria | Median (IQR) | IQR interpretation | # of Panellists |
| --- | --- | --- | --- |
| End users’ background | 5 (4,5) | Fair Consensus | 24/25 |
| Easiness of use | 4 (4,5) | Fair Consensus | 24/25 |
| Training needs | 4 (4,5) | Fair Consensus | 25/25 |
| User's understanding of the technical and clinical impact | 4 (3,5) | Sufficient Consensus | 25/25 |
| Installation requirements | 5 (4,5) | Fair Consensus | 25/25 |
| Maintenance frequency | 4 (4,5) | Fair Consensus | 25/25 |
| Maintenance complexity | 4 (4,5) | Fair Consensus | 25/25 |
| Need for consumables | 5 (4,5) | Fair Consensus | 25/25 |
| Need for spare parts | 5 (4,5) | Fair Consensus | 25/25 |
| Compatible consumables/spare parts | 4 (3,5) | Sufficient Consensus | 25/25 |
| Portability, compactness, robustness | 5 (4,5) | Fair Consensus | 25/25 |
| Limiting the number of components/spare parts | 4 (4,5) | Fair Consensus | 25/25 |
| Reusability | 4 (3,5) | Sufficient Consensus | 25/25 |
| Reliance on power sources | 4 (4,5) | Fair Consensus | 25/25 |
| Reliance on water distribution | 4 (3,5) | Sufficient Consensus | 24/25 |
| Reliance on medical location air | 4 (3,4) | Fair Consensus | 25/25 |
| Understanding/stating the dependence of the md from the medical location characteristics | 3 (3,4) | Fair Consensus | 20/25 |
| Need for sample preparation | 4 (3,4) | Fair Consensus | 24/25 |
| Robustness of the material | 4 (4,5) | Fair Consensus | 25/25 |
| Durability of the material | 4 (4,5) | Fair Consensus | 25/25 |
| Initial cost | 4 (4,5) | Fair Consensus | 25/25 |
| Maintenance costs | 5 (5,5) | Full consensus | 25/25 |
| Running costs | 5 (4,5) | Fair Consensus | 25/25 |
| MD lifetime | 4 (4,5) | Fair Consensus | 25/25 |
| Lifetime of MD parts/components | 4 (4,5) | Fair Consensus | 25/25 |
